# Supplementary material for: Knowledge, attitudes, and fear of COVID-19 during the Rapid Rise Period in Bangladesh
Source: PLoS One. 2020 Sep 24;15(9):e0239646. doi: 10.1371/journal.pone.0239646 (PMC7514023; doi:10.1371/journal.pone.0239646)
Supplement: S1 File — (DOCX) [file pone.0239646.s001.docx]

**Knowledge, attitudes, and Fear of COVID-19 during the rapid rise period in Bangladesh**

**Part 1: Socio-demographics**

| Q | Question | Variables/ Instruction | Answer |
| --- | --- | --- | --- |
| 1 | Date | Put date |  |
| 2 | Name | write/ patient can hide name |  |
| 3 | Village/ street/ address | write |  |
| 4 | Police Station | write |  |
| 5 | District: | write |  |
| 7 | Gender | 1) Male, 2) Female |  |
| 8 | Age | write |  |
| 9 | Education | Write highest educational level |  |
| 10 | Travel last 14 days? | 1) yes, 2) no, if yes write place |  |
| 11 | Fever >100F in last 14 days | 1) yes, 2) no, if yes write duration and highest degree F |  |
| 12 | Shortness of Breath in last 14 days | 1) yes, 2) no, if yes duration in days |  |
| 13 | Dry cough in last 14 days | 1) yes, 2) no, if yes duration in days |  |
| 14 | Sore throat in last 14 days | 1) yes, 2) no, if yes duration in days |  |
| 15 | Cough with sputum in last 14 days | 1) yes, 2) no, if yes duration in days |  |
| 16 | Pneumonia in last 14 days | 1) yes, 2) no, if yes duration in days |  |
| 17 | Diarrhoea in last 14 days | 1) yes, 2) no, if yes duration in days |  |
| 18 | Anosmia or Taste loss | 1) yes, 2) no, if yes duration in days |  |
| 19 | Arthralgia in last 14 days | 1) yes, 2) no, if yes duration in days |  |
| 20 | Conjunctivitis in last 14 days | 1) yes, 2) no, if yes duration in days |  |
| 21 | Fatigue in last 14 days | 1) yes, 2) no, if yes duration in days |  |
| 22 | Malaise in last 14 days | 1) yes, 2) no, if yes duration in days |  |
| 23 | Other | 1) yes, 2) no, if yes specify |  |
| 24 | Hospitalized in last 14 days | 1) yes, 2) no, if yes diagnosis & duration |  |
| 25 | Risk: Diabetes | 1) yes, 2) no, if yes duration in months |  |
| 26 | COPD or Lung disease | 1) yes, 2) no, if yes duration in months |  |
| 27 | Heart disease | 1) yes, 2) no, if yes duration in months |  |
| 28 | Liver disease | 1) yes, 2) no, if yes duration in months |  |
| 29 | Neurological disease/ disability | 1) yes, 2) no, if yes duration in months |  |
| 30 | Chronic MSK problem | 1) yes, 2) no, if yes duration in months |  |
| 31 | Kidney disease | 1) yes, 2) no, if yes duration in months |  |
| 32 | Smoking | 1) yes, 2) no, if yes duration in months |  |
| 33 | Others | 1) yes, 2) no, if yes specify and duration in months |  |
| 34 | Occupation: Public service | 1) yes, 2) no |  |
| 35 | Occupation: healthcare | 1) yes, 2) no |  |
| 36 | Occupation: Business or work in crowd | 1) yes, 2) no |  |
| 37 | Study in school/ college/ university | 1) yes, 2) no |  |
| 38 | Diagnosed COVID 19 in family/ community/work/ education | 1) yes, 2) no |  |

**Part 2: Knowledge**

| 1 | The main clinical symptoms of COVID-19 are fever, fatigue, dry cough, and myalgia. | 1)True, 2)false, 3)I don’t know |
| --- | --- | --- |
| 2 | Stuffy nose, runny nose, and sneezing are less common in COVID-19. | 1)True, 2)false, 3)I don’t know |
| 3 | Currently is no effective cure but early symptomatic and supportive treatment can help. | 1)True, 2)false, 3)I don’t know |
| 4 | Not all COVID-2019 patients develop to severe cases, elderly, chronic illnesses, and obesity are likely to be severe. | 1)True, 2)false, 3)I don’t know |
| 5 | Eating or contacting wild animals would result in the infection by the COVID-19 virus. | 1)True, 2)false, 3)I don’t know |
| 6 | Persons with COVID-2019 cannot infect the virus to others when a fever is not present. | 1)True, 2)false, 3)I don’t know |
| 7 | The COVID-19 virus spreads via respiratory droplets of infected individuals. | 1)True, 2)false, 3)I don’t know |
| 8 | Ordinary residents can wear general medical masks to prevent the infection by the COVID-19 virus. | 1)True, 2)false, 3)I don’t know |
| 9 | It is not necessary for children and young adults to take measures to prevent the infection by the COVID-19 virus. | 1)True, 2)false, 3)I don’t know |
| 10 | To prevent the infection by COVID-19,we should avoid going to crowded places and avoid taking public transportations. | 1)True, 2)false, 3)I don’t know |
| 11 | Isolation and treatment of people infected with the COVID-19 virus are effective ways to reduce the spread of virus | 1)True, 2)false, 3)I don’t know |
| 12 | People who have contact with someone infected with the COVID-19 virus should be immediately isolated for 14 days. | 1)True, 2)false, 3)I don’t know |

**Part 3: Attitudes**

| 1. Do you agree that COVID-19 will finally be successfully controlled?  2. Do you have confidence that Bangladesh can win the battle against the COVID-19 virus? | 1)Agree, 2) disagree, 3) I don’t know |
| --- | --- |
|  | 1) Yes, 2) No, 3) Don't know |

**Part 4: Practices**

| 1. In last 7 days, have you gone to any crowded place? | 1) Yes, 2)no |
| --- | --- |
| 2. In last 7 days, have you worn a mask when leaving home? | 1) Yes, 2)no |

**Part 5: Fear of COVID 19**

| 1.I am most afraid of Coronavirus-19 | 1=strongly disagree | 4=agree |
| --- | --- | --- |
|  | 2=disagree | 5=strongly agree |
|  | 3=neither agree nor |  |
| 2. It makes me uncomfortable to think about coronavirus-19 | 1=strongly disagree | 4=agree |
|  | 2=disagree | 5=strongly agree |
|  | 3=neither agree nor |  |
| 3. My hands become clammy when I think about coronavirus-19 | 1=strongly disagree | 4=agree |
|  | 2=disagree | 5=strongly agree |
|  | 3=neither agree nor |  |
| 4. I am afraid of losing my life because of coronavirus-19 | 1=strongly disagree | 4=agree |
|  | 2=disagree | 5=strongly agree |
|  | 3=neither agree nor |  |
| 5. When watching news and stories about coronavirus-19 on social media, I become nervous or anxious. | 1=strongly disagree | 4=agree |
|  | 2=disagree | 5=strongly agree |
|  | 3=neither agree nor |  |
| 6. I cannot sleep because I’m worrying about getting coronavirus-19. | 1=strongly disagree | 4=agree |
|  | 2=disagree | 5=strongly agree |
|  | 3=neither agree nor |  |
| 7. My heart races or palpitates when I think about getting coronavirus-19 | 1=strongly disagree | 4=agree |
|  | 2=disagree | 5=strongly agree |
|  | 3=neither agree nor |  |
