# Supplementary material for: Knowledge, attitudes, and fear of COVID-19 during the Rapid Rise Period in Bangladesh
Source: PLoS One. 2020 Sep 24;15(9):e0239646. doi: 10.1371/journal.pone.0239646 (PMC7514023; doi:10.1371/journal.pone.0239646)
Supplement: S2 File — (DOCX) [file pone.0239646.s002.docx]

**"Knowledge, attitudes, and Fear of COVID-19 during the rapid rise period in Bangladesh"**

**অংশ১- জনসংখ্যা তত্বীয় তথ্যাবলী**

| **প্রশ্ন** | **নির্দেশনা** | | **উত্তর** |
| --- | --- | --- | --- |
| ১. তারিখ | লিখুন | |  |
| ২. নাম | লিখুন | |  |
| ৩. গ্রাম/ রাস্তা/ ঠিকানা | লিখুন | |  |
| ৪. থানা | লিখুন | |  |
| ৫. জেলা | লিখুন | |  |
| ৬. লিঙ্গ | ১=পুরুষ | ২=মহিলা |  |
| ৭. বয়স | লিখুন | |  |
| ৮. শিক্ষাগত যোগ্যতা | সর্বোচ্চ শিক্ষালিখুন | |  |
| ৯. গত ১৪ দিন দেশের বাইরে গিয়েছেন/ এসেছেন? | ১= হ্যাঁ  ২= না | | হ্যাঁ হলে, জায়গার নাম লিখুন.............. |
| ১০. গত ১৪ দিনে জ্বর ১০০ফা. এর উপরে উঠেছে? | ১= হ্যাঁ  ২= না | | হ্যাঁ হলে, সর্বোচ্চ ফা. লিখুন |
| ১১. গত ১৪ দিনে শ্বাস কস্ট ছিল? | ১= হ্যাঁ  ২= না | | হ্যাঁ হলে, কতদিন লিখুন |
| ১২. গত ১৪ দিনে শুকনা কাশি ছিল? | ১= হ্যাঁ  ২= না | | হ্যাঁ হলে, কতদিন লিখুন |
| ১৩. গত ১৪ দিনে গলা ব্যথাছিল? | ১= হ্যাঁ  ২= না | | হ্যাঁ হলে, কতদিন লিখুন |
| ১৪. গত ১৪ দিনে কফ সহ কাশি ছিল? | ১= হ্যাঁ  ২= না | | হ্যাঁ হলে, কতদিন লিখুন |
| ১৫. গত ১৪ দিনে নিউমোনিয়া ছিল? | ১= হ্যাঁ  ২= না | | হ্যাঁ হলে, কতদিন লিখুন |
| ১৬. গত ১৪ দিনে কি ডাইরিয়া ছিল? | ১= হ্যাঁ  ২= না | | হ্যাঁ হলে, কতদিন লিখুন |
| ১৭. গত ১৪ দিনে কি স্বাদ ঘ্রাণ অনুভব করেননি? | ১= হ্যাঁ  ২= না | | হ্যাঁ হলে, কতদিন লিখুন |
| ১৮. গত ১৪ দিনে জয়েন্ট ব্যথা ছিল? | ১= হ্যাঁ  ২= না | | হ্যাঁ হলে, কতদিন লিখুন |
| ১৯.গত ১৪ দিনে কি চোখ উঠেছে? | ১= হ্যাঁ  ২= না | | হ্যাঁ হলে, কতদিন লিখুন |
| ২০. গত ১৪ দিনে কি শারিরীক দূর্বলতা অনুভব করেছেন? | ১= হ্যাঁ  ২= না | |  |
| ২১. গত ১৪ দিনে কি আপনার গা ম্যাজ ম্যাজ করেছে ? | ১= হ্যাঁ  ২= না | |  |
| ২২. গত ১৪ দিনে অন্য কোন শারিরিক অসুস্থতা ছিল? | ১= হ্যাঁ  ২= না | |  |
| ২৩. গত ১৪ দিনে হাসপাতালে ভর্তি ছিল? | ১= হ্যাঁ  ২= না | | হ্যাঁ হলে, ডায়াগনোসিস ও সময় লিখুন |
| ২৪. আপনার কি ডায়াবেটিস আছে? | ১= হ্যাঁ  ২= না | | হ্যাঁ হলে, কত মাস যাবত লিখুন |
| ২৫. আপনার সি ও পি ডি বা হাঁপানি আছে? | ১= হ্যাঁ  ২= না | | হ্যাঁ হলে, কত মাস যাবত লিখুন |
| ২৬. আপনার হৃদরোগ আছে? | ১= হ্যাঁ  ২= না | | হ্যাঁ হলে, কত মাস যাবত লিখুন |
| ২৭. আপনার লিভারের রোগ আছে? | ১= হ্যাঁ  ২= না | | হ্যাঁ হলে, কত মাস যাবত লিখুন |
| ২৮. আপনার স্ট্রোক বা পক্ষাঘাত বা প্রতিবন্ধিতা আছে? | ১= হ্যাঁ  ২= না | | হ্যাঁ হলে, কত মাস যাবত লিখুন |
| ২৯. আপনার দীর্ঘদিনের বাতব্যথা আছে? | ১= হ্যাঁ  ২= না | | হ্যাঁ হলে, কত মাস যাবত লিখুন |
| ৩০. আপনার কিডনী রোগ আছে? | ১= হ্যাঁ  ২= না | | হ্যাঁ হলে, কত মাস যাবত লিখুন |
| ৩১. আপনি ধুমপান করেন? | ১= হ্যাঁ  ২= না | | হ্যাঁ হলে, কত মাস যাবত লিখুন |
| ৩২. আপনি কি অন্য কোন রোগে আক্রান্ত বা চিকিৎসা নিচ্ছেন? | ১= হ্যাঁ  ২= না | | হ্যাঁ হলে, কত মাস যাবত লিখুন |
| ৩৩. আপনি কি পাবিলিক সার্ভিস করেন? | ১= হ্যাঁ  ২= না | |  |
| ৩৪. আপনি কি চিকিৎসা পেশাজীবী? | ১= হ্যাঁ  ২= না | |  |
| ৩৫. আপনি কি জনাকীর্ণ স্থানে কাজ বা ব্যবসা করেন? | ১= হ্যাঁ  ২= না | |  |
| ৩৬. আপনি কি স্কুল, কলেজবা বিশ্ববিদ্যালয়ে পড়েন? | ১= হ্যাঁ  ২= না | |  |
| ৩৭. আপনার পরিবার, এলাকা, কর্মক্ষেত্রে বা জানা কারো কি কোভিড ১৯ ধরা পড়েছে? | ১= হ্যাঁ  ২= না | |  |

**অংশ২- কোভিড ১৯ সংক্রান্ত জ্ঞান**

| ১. কোভিড ১৯ এর প্রধান লক্ষণ হল জ্বর, দূর্বলতা, শুকনা কাশি ও শরীর ব্যথা | ১= সত্য | ৩= জানিনা |
| --- | --- | --- |
|  | ২= মিথ্যা |  |
| ২. নাক বন্ধ, নাকে সর্দি, হাঁচি কোভিড ১৯ এ কম হয় | ১= সত্য | ৩= জানিনা |
|  | ২= মিথ্যা |  |
| ৩. কোভিড ১৯ এর সরাসরি কোন চিকিৎসা নেই তবে প্রথমেই লক্ষণ অনুযায়ী চিকিৎসা ও সাপোর্টিভ চিকিৎসা উপকারী | ১= সত্য | ৩= জানিনা |
|  | ২= মিথ্যা |  |
| ৪. সব কোভিড ১৯ রোগীরঅবস্থা জটিল হয় না, বয়স্ক, দীর্ঘদিনের অসুস্থতা আর ওবিসিটি রোগীদের অবস্থা খারাপ হতে পারে | ১= সত্য | ৩= জানিনা |
|  | ২= মিথ্যা |  |
| ৫. বণ্য জীবজন্তু খাওয়া বা স্পর্শ করলে কোভিড ১৯ ভাইরাসে আক্রান্ত হতে পারে | ১= সত্য | ৩= জানিনা |
|  | ২= মিথ্যা |  |
| ৬. কোভিড ১৯ আক্রান্ত ব্যক্তির জ্বর না থাকলে এ রোগ ছড়াতে পারে না | ১= সত্য | ৩= জানিনা |
|  | ২= মিথ্যা |  |
| ৭. কোভিড ১৯ আক্রান্ত ব্যাক্তির হাঁচি কাশি কফ থেকে ছড়াতে পারে | ১= সত্য | ৩= জানিনা |
|  | ২= মিথ্যা |  |
| ৮. সাধারন মানুষ মেডিক্যাল মাস্ক পরে এ সংক্রমণ প্রতিরোধ করতে পারে | ১= সত্য | ৩= জানিনা |
|  | ২= মিথ্যা |  |
| ৯. শিশু ও কম বয়সী যুবাদের কোভিড ১৯ সংক্রমণ প্রতিরোধে ব্যবস্থা নেয়ার প্রয়োজন নেই | ১= সত্য | ৩= জানিনা |
|  | ২= মিথ্যা |  |
| ১০. কোভিড ১৯ সংক্রমণ প্রতিরোধে জনাকীর্ণ স্থান বাপাবলিক ট্রান্সপোর্ট পরিহার করতে হবে | ১= সত্য | ৩= জানিনা |
|  | ২= মিথ্যা |  |
| ১১. কোভিড ১৯ আক্রান্ত ব্যাক্তিকে আলাদা করে চিকিৎসা করলে এ সংক্রমণ প্রতিরোধ করা যায় | ১= সত্য | ৩= জানিনা |
|  | ২= মিথ্যা |  |
| ১২. কেউ কোভিড ১৯ আক্রান্ত ব্যক্তির সংস্পর্শে আসলে তাকে তাৎক্ষনিক ১৪ দিনের জন্য আলাদা করা উচিত | ১= সত্য | ৩= জানিনা |
|  | ২= মিথ্যা |  |

**অংশ৩- কোভিড ১৯ সংক্রান্ত মনোভাব**

| ১. আপনি কি মনে করেন কোভিড ১৯ শেষ পর্যন্ত কন্ট্রোল করা যাবে? | ১=একমত | ৩=জানি না |
| --- | --- | --- |
|  | ২=দ্বিমত |  |
| ২. আপনি কি নিশ্চিত যে বাংলাদেশ কোভিড ১৯ এর বিরুদ্ধে যুদ্ধে জিততে পারবে? | ১=হ্যাঁ | ৩=জানি না |
|  | ২=না |  |

**অংশ৪- কোভিড ১৯ সংক্রান্ত অনুশীলন**

| ১. আপনি কি গত ৭ দিনে জনাকীর্ণ স্থানে গিয়েছেন? | ১= হ্যাঁ  ২= না |
| --- | --- |
| ২. আপনি কি গত ৭ দিনে ঘর থেকে বের হবার সময় মাস্ক পরেছেন? | ১= হ্যাঁ  ২= না |

**অংশ৫- কোভিড ১৯ সংক্রান্ত ভয়**

| ১. আমি প্রায়ই করোনা ভাইরাস ১৯ এর ভয়ে ভীত থাকি | ১=একদমই দ্বিমত | ৪= একমত |
| --- | --- | --- |
|  | ২=দ্বিমত | ৫=একদমত একমত |
|  | ৩= একমত বা দ্বিমত কোনটিই নই | |
| ২. করোনা ভাইরাস ১৯ সম্পর্কে চিন্তা করতে আমার আস্বস্তি লাগে | ১=একদমই দ্বিমত | ৪= একমত |
|  | ২=দ্বিমত | ৫=একদমত একমত |
|  | ৩= একমত বা দ্বিমত কোনটিই নই | |
| ৩. করোনা ভাইরাস ১৯ এর কথা ভাবলেই আমার হাত ঘামাতে থাকে | ১=একদমই দ্বিমত | ৪= একমত |
|  | ২=দ্বিমত | ৫=একদমত একমত |
|  | ৩= একমত বা দ্বিমত কোনটিই নই | |
| ৪. করোনা ভাইরাস ১৯ এ আক্রান্ত হয়ে আমার জীবন হারানোর ভয় হয় | ১=একদমই দ্বিমত | ৪= একমত |
|  | ২=দ্বিমত | ৫=একদমত একমত |
|  | ৩= একমত বা দ্বিমত কোনটিই নই | |
| ৫. করোনা ভাইরাস ১৯ এর খবর ও গল্প দেখতে ও সোশ্যাল মিডিয়ায় শুনতেই আমি নার্ভাস বা উদ্বিগ্ন হয়েপড়ি | ১=একদমই দ্বিমত | ৪= একমত |
|  | ২=দ্বিমত | ৫=একদমত একমত |
|  | ৩= একমত বা দ্বিমত কোনটিই নই | |
| ৬. করোনা ভাইরাস ১৯ এ আকন্ত হবার চিন্তায় আমার ঘুম আসেনা | ১=একদমই দ্বিমত | ৪= একমত |
|  | ২=দ্বিমত | ৫=একদমত একমত |
|  | ৩= একমত বা দ্বিমত কোনটিই নই | |
| ৭. করোনা ভাইরাস ১৯ এ আক্রান্ত হবার চিন্তায় আমার বুক ধড়ফড় করে ও ধুকধুক করে | ১=একদমই দ্বিমত | ৪= একমত |
|  | ২=দ্বিমত | ৫=একদমত একমত |
|  | ৩= একমত বা দ্বিমত কোনটিই নই | |
