# Supplementary material for: Knowledge, attitudes, and fear of COVID-19 during the Rapid Rise Period in Bangladesh
Source: PLoS One. 2020 Sep 24;15(9):e0239646. doi: 10.1371/journal.pone.0239646 (PMC7514023; doi:10.1371/journal.pone.0239646)
Supplement: Attachment — Submitted filename: Respose to reviewers.docx [file pone.0239646.s003.docx]

**Response to Reviewers**

Dear Reviewers

Thank you for your comments. We appreciate your evaluation of our work and the manuscript. Please find our responses.

| Reviewers comment | Authors response |
| --- | --- |
| The study entitled “Knowledge, Attitudes, and Fear of COVID-19 during the Rapid Rise Period in Bangladesh” has the strength of a large sample size (n=2157). However, the manuscript suffers from many flaws and I am not sure whether the authors can satisfactorily revise their work | Thank you for your positive comments; we have revised the manuscript substantially. |
| 1. The authors have wrong statistical knowledge in the significance level. We do not use p<=0.05, we use p<0.05. That is, if the p-value is right at the 0.05, the comparison is not significant. However, the authors use <= throughout the manuscript | We agree, we put p=<.05 in the manuscript to reflect “p is equal to less than point zero five”, we didn’t put it after the less symbol, but it is creating confusion, so we corrected to p<.05 |
| 2. The authors conducted some ANOVA tests and did not further conduct the post-hoc analysis | Agreed, We excluded the ANOVA to examine the association between categorical and numerical data and placed with a “Chi-square test” as per statistician’s suggestion. <https://pubmed.ncbi.nlm.nih.gov/23894860/> |
| 3. The authors used “r” to report the regression findings. However, “r” refers to Pearson’s’ r (or some use an r to represent Spearman’s rho). The r should not be used for regression models and the presentation is confusing and misleading. | We agreed and have made that change. |
| 4. The authors sometimes report a p-value at 0.000. However, it is impossible for a p-value to be exactly at 0.000. p<0.001 should be used instead | Agreed, in some cases, the value is .00001, so we turned to .000, but accepted the suggestion and changed to .0001 or .001 |
| 5. The information of questions on knowledge, attitude, and practice is vague and insufficient for me to evaluate. Specifically, the psychometric properties of these questions are unknown and how did the authors ensure that these questions developed from a Chinese study can be applicable to the present study? | Questions related to KAP adapted from the survey questions used in a study conducted during a period of rapid case increases in China (<https://www.ncbi.nlm.nih.gov/pmc/articles/PMC7098034/> ). The KAP section of the questionnaire related to a total of 12 score knowledge questions on COVID-19, categorical answers to attitudes towards the control of the pandemic, and practices of wearing masks and avoiding public gatherings. The questionnaire had a wider acceptance and was retrieved by a couple of good quality papers in the same geographic area. Some papers used the same questionnaire in Malaysia (<https://journals.plos.org/plosone/article?id=10.1371/journal.pone.0233668> ), in Egypt (<https://link.springer.com/content/pdf/10.1007/s10900-020-00827-7.pdf> ), and in Saudi Arabia (<https://www.ncbi.nlm.nih.gov/pmc/articles/PMC7266869/> ).  The questionnaire complied with the forward and back-translation into Bangla by a bilingual British researcher and sent to two renounced bilingual epidemiologists in Bangladesh to examine the difference and suitability of the questionnaire. Also, a pilot study was conducted before the commencement of the research. This information we added in the method section for better clarification.  Moreover, the Fear of COVID-19 Scale (FCV 19S) was used and the Bangla version reported valid and reliable tool with robust psychometric properties (see the link, <https://link.springer.com/article/10.1007/s11469-020-00270-8> ). The psychometric properties of the Bangla version KAP not yet published. However, as mentioned earlier, the forward and back-translation into Bangla as well as a pilot study justified the use of KAP.  Also, the questions were the best fit for our geography, study objectives, and similarity of rapid raise situation. |
| 6. The abbreviation of the Fear of COVID-19 Scale should be FCV-19S, not FCS. Also, the psychometric properties paper on Bangla version of the FCV-19S has been published. The authors should acknowledge this and cite properly | Agreed and revised. We couldn’t use the Bangla version with psychometric validated question because it has been published in May 2020 <https://www.ncbi.nlm.nih.gov/pmc/articles/PMC7213549/>  And we started data collection in April 2020. |
| 7. The Introduction does not prepare the readers why the authors want to examine knowledge, attitude, practice, and fear for different subgroups using the demographics. Also, it is unclear why the authors wanted to examine the associations between these factors. | Agreed, and revised in Page 4, as  Determining knowledge, attitudes, practices (KAP), and fear will provide a glimpse of how Bangladesh is responding to the pandemic in this state of rising cases. This will further help to evaluate their overall preparedness. The COVID-19 crisis is assumed to be a long-term process, and the only way to battle the pandemic is to know the right information and practice the recommended health advisories. It is also necessary to examine the relationship among demographic variables with KAP and fear to explore the in-depth understanding of factors contributing to the preparedness for and response towards COVID-19. |
| 8. Line 207, I cannot understand why the people aged between 41 and 60 years can be defined as “young adults”. They are already middle age. Also, what is the meaning of “young” here? Do the authors want to say youth? | Omitted, kept only Adult  We tried to chronologically classify as  Adolescents  Youth  Young Adult (converted to Adult)  Elderly |
| 9. The authors reported the COVID 19 Symptoms and Co-morbidity. However, the authors did not mention how they assessed these symptoms and co-morbidity. | Discussed in the Methodology section in Questionnaire headings, Page 4-5, line (130-133) as  The co-morbidities and symptoms of COVID-19 were obtained from WHO resources and asked to the respondents’ whether present or not <https://www.who.int/docs/default-source/coronaviruse/situation-reports/20200309-sitrep-49-covid-19.pdf?sfvrsn=70dabe61_4>  Explanation  The symptom list is taken from the mentioned document page 9, how to suspect a case  Co-morbidities was taken from <https://erj.ersjournals.com/content/early/2020/03/17/13993003.00547-2020> |
| 10. The authors mentioned that they used chi-square test in the Data analysis section; however, there are no results on chi-square values in the Results section. | Agreed, added in Table 3 |
| 11. The presentation of Table 2 is confusing. What are the “others” in the variables column? Also, the authors should explicitly indicate what the reference group is. | Table 2 statistics has been changed and defined the others |
| 12. The authors mentioned that they performed binary logistic regression in the Data analysis section; however, I see no results on the logistic regression in the Results section. | Agreed, the analysis reformed and tested as binary logistic regression with the concern of statistician. Putted Chi-square result, Co-efficient result, Odd ratio value, and p-value |
| 13. The meanings of SSC and HSC in the Table 3 are unclear. | Agreed, put in the table and narrated bellow as secondary school certificate and higher secondary school certificate |
| 14. The Discussion reports redundant information; that is, the author repeated their results in the Discussion again. However, I did not see the authors “explain” their findings in the Discussion. They simply repeated the Results and then mentioned other similar studies’ findings. | Agreed and revised in each finding.  Page 15, line 338-343  Page 15 line 354-358  Page 16 line 372-374  Page 16 line 378-384  Also, it is difficult to explain why the study findings are such, because this is a cross-sectional study and focused on observation and relationship. Any comment on why needs in-depth findings that are missing in this field. Hence we tried best to explain. |
| 15. There are some awkward sentences hinder the reading. For example, “Limitations response rates, correlation with fear and psychological issues, and completion of the questionnaire for COVID-19 positive. We recommend future studies to include information on limitation of movement, isolation and insomnia as it relates to psychological illness, as well as information regarding neurological signs and symptoms of patients and relationship to cognition and fear.” | We have prepared the manuscript with native English speakers; hence we agree the language can be better. We supplied the manuscript to PLOS recommended authorship services to make fit and appropriate with your comments. The language editing has been performed by professional scientific editing service AJE recommended by PLOS.  We have revised the paragraph as-  Limitations included the response rate (62.85%) and the completion of the questionnaire by none COVID-19-positive individuals. We recommend that future studies include information on the long-term observation of corvid-19-positive cases or cases with symptoms with respect to movement, function, physical signs, mental health, and quality-of-life issues. |
| 16. The authors mentioned the age range of 13-90 in the Abstract. However, the age range is 13-88 in the Results section. | Apology, it was unintentional. Our eligibility criteria were 13-90 years but we found 13- 88 years. Corrected in the abstract. |

Additional requirements

| Additional requirements | Authors response |
| --- | --- |
| 1. Please ensure that your manuscript meets PLOS ONE's style requirements, including those for file naming  2. We suggest you thoroughly copyedit your manuscript for language usage, spelling, and grammar. If you do not know anyone who can help you do this, you may wish to consider employing a professional scientific editing service. | To ensure the paper fits best with the language and journal format, we sent the paper in your recommended professional scientific editing service. The language editing has been performed by professional scientific editing service AJE recommended by PLOS. |
| 3. Please include additional information regarding the survey or questionnaire used in the study and ensure that you have provided sufficient details that others could replicate the analyses. For instance, if you developed a questionnaire as part of this study and it is not under a copyright more restrictive than CC-BY, please include a copy, in both the original language and English, as Supporting Information. Moreover, please include more details on how the questionnaire was pre-tested, and whether it was validated | Supplied as additional information- questionnaire  The supplied files are  **S1 Table. Table 1.** Relationship of demographic characteristics with Knowledge and fear  **S2 Table. Table 2.** Results of Binary logistic regression on factors associated with Knowledge and fear  **S3 Table. Table 3.** The relationship among Attitude and practice with demographic variables  **S1 File. Track changed Manuscript for professional scientific editing service.** The track changing enabled by AJE professional scientific editing services recommended by PLOS one (<http://learn.aje.com/plos/>)  **S2 File. English Questionnaire**  **S3 File. Bangla Questionnaire** |
| 4. In your Methods section, please provide additional information about the participant recruitment method and the demographic details of your participants. Please ensure you have provided sufficient details to replicate the analyses such as: a) the recruitment date range (month and year), b) a description of any inclusion/exclusion criteria that were applied to participant recruitment, c) a table of relevant demographic details, d) a statement as to whether your sample can be considered representative of a larger population, e) a description of how participants were recruited, and f) descriptions of where participants were recruited and where the research took place. | Supplied and mentioned each part (a-f) from Page 4- line 117 to page 5-line 153. |
| 5. We noted in your submission details that a portion of your manuscript may have been presented or published elsewhere. The article is in the MedRxiv pre-print server. Please clarify whether this publication was peer-reviewed and formally published. If this work was previously peer-reviewed and published, in the cover letter please provide the reason that this work does not constitute dual publication and should be included in the current manuscript | It was a pre-print server and it was saying that your journal allows the pre-print server (<https://www.medrxiv.org/submit-a-manuscript>). If the paper is accepted and published in your journal, the crossref and doi will be automatically updated. The revised manuscript can be preprinted also.  Our pre-print manuscript <https://www.medrxiv.org/content/10.1101/2020.06.17.20133611v1> |
| 6. Your ethics statement must appear in the Methods section of your manuscript. If your ethics statement is written in any section besides the Methods, please move it to the Methods section and delete it from any other section. Please also ensure that your ethics statement is included in your manuscript, as the ethics section of your online submission will not be published alongside your manuscript | We put in Method section only on page 5 line 139 to line 143 |
